# Supplementary material for: Number of simultaneously acting global change factors affects composition, diversity and productivity of grassland plant communities
Source: Nat Commun. 2022 Dec 19;13:7811. doi: 10.1038/s41467-022-35473-1 (PMC9763497; doi:10.1038/s41467-022-35473-1)
Supplement: Supplementary file 1 — Supplementary information file [file 41467_2022_35473_MOESM1_ESM.pdf]

Supporting information for:

Number of simultaneously acting global change factors affects plant species composition, diversity and productivity of grassland communities

Benedikt Speißer[1]*, Rutger A. Wilschut[1,2], Mark van Kleunen[1,3]

[1]*Ecology, Department of Biology, University of Konstanz, 78464 Konstanz, Germany*

[2]*Department of Nematology, Wageningen University and Research, 6708 PB Wageningen, The Netherlands*

[3]*Zhejiang Provincial Key Laboratory of Plant Evolutionary Ecology and Conservation, Taizhou University, Taizhou 318000, China*

*Contact information corresponding author:

Mail: benedikt.speisser@uni-konstanz.de

Phone: +49 7531 4145

Supplementary Table 1 Individual GCF effects on the transplanted-seedling community. P-values for effects of single-GCF treatments in the transplanted-seedling community, obtained from the model summaries (two-sided t-tests, without adjustments for multiple comparisons).

Treatment	Total aboveground biomass	PC1 species proportions	PC2 species proportions	Diversity species proportions	Evenness species proportions
Fungicide	0.129	0.530	0.595	0.878	0.942
Light pollution	0.740	0.819	0.487	0.209	0.219
Microplastic	**0.048***	0.853	0.569	0.970	0.968
Eutrophication	**< 0.001**	0.729	*0.095*	0.157	0.117
Salinization	**< 0.001**	0.607	**0.035**	0.622	0.609
Warming	0.176	0.363	0.729	0.214	0.445

*Significant effects ($p < 0.05$) are shown in bold, marginally significant effects ($p < 0.1$) in italics.

Supplementary Table 2 Individual GCF effects on the sown community. P-values for effects of single-GCF treatments in the sown community, obtained from the model summaries (two-sided t-tests, without adjustments for multiple comparisons).

Treatment	Total aboveground biomass	PC1 species proportions	PC2 species proportions	Diversity species proportions	Evenness species proportions
Fungicide	0.564	0.712	0.611	0.327	0.989
Light pollution	0.488	0.351	0.347	0.495	0.494
Microplastic	**0.026***	**< 0.001**	0.226	0.685	0.756
Eutrophication	**< 0.001**	*0.093*	**0.008**	0.400	0.210
Salinization	*0.065*	**0.001**	0.997	0.162	0.300
Warming	0.543	**0.001**	0.206	0.930	0.560

*Significant effects (p < 0.05) are shown in bold, marginally significant effects (p < 0.1) in italics.

Supplementary Table 3 Effects of single GCFs and GCF number on the sown communities. Results of linear (mixed effects) models testing the effects of the single GCF treatments (a) and GCF number (b) on aboveground biomass, species composition (PC1 and PC2 of PCA on species biomass proportions), diversity (Shannon index) and evenness of the sown communities. Significance of the fixed effects was assessed using log-likelihood ratios (χ^2 values) or F statistics, as indicated in the table.

		Total aboveground community biomass		PC1 species proportions		PC2 species proportions		Shannon index (species prop.)		Pielou evenness (species prop.)	
						(a) GCF-treatment model					
Fixed effects	df	χ^2	p*	χ^2	p	χ^2	p	F	p	χ^2	p
GCF treatment	6	95.10	**< 0.001**	64.94	**< 0.001**	18.93	**0.004**	1.22	0.32	6.69	0.35
Random effects		SD		SD		SD		SD		SD	
Plot		0.0774		0.0902		0.1216		–		0.0342	
Residual		0.2943		0.1399		0.1837		–		0.1021	
						(b) GCF-number model					
Fixed effects	df	χ^2	p	χ^2	p	χ^2	p	χ^2	p	F	p
GCF number	1	2.62	0.11	0.30	0.58	15.10	**< 0.001**	36.01	**< 0.001**	23.54	**< 0.001**
Random effects		SD		SD		SD		SD		SD	
Plot		0.0612		–		–		–		–	
GCF combination		0.5025		0.0826		0.0558		0.0783		–	
Residual		0.2976		0.1892		0.1827		0.2652		–	

*Bold p values indicate significant effects ($p < 0.05$).

Supplementary Table 4 Effects of single GCFs and GCF number on the transplanted-seedling communities. Results of linear (mixed effects) models testing the effects of the single GCF treatments (a) and GCF number (b) on aboveground biomass, species composition (PC1 and PC2 of PCA on species biomass proportions), diversity (Shannon index) and evenness of the transplanted-seedling communities. Significance of the fixed effects was assessed using log-likelihood ratios (χ^2 values) or F statistics, as indicated in the table.

	Total aboveground community biomass			PC1 species proportions		PC2 species proportions		Shannon index (species prop.)		Pielou evenness (species prop.)	
					(a) GCF-treatment model						
Fixed effects	df	χ^2	p*	χ^2	p	χ^2	p	F	p	F	p
GCF treatment	6	600.58	**< 0.001**	2.99	0.81	7.13	0.31	0.96	0.46	1.18	0.34
Random effects		SD		SD		SD		SD		SD	
Plot		0.7832		0.1026		0.0819		–		–	
Residual		2.2939		0.1685		0.1738		–		–	
					(b) GCF-number model						
Fixed effects	df	χ^2	p	χ^2	p	χ^2	p	χ^2	p	χ^2	p
GCF number	1	4.74	**0.029**	2.33	0.13	5.59	**0.018**	16.32	**< 0.001**	14.58	**< 0.001**
Random effects		SD		SD		SD		SD		SD	
Plot		0.1984		0.0859		0.0234		0.0564		0.0161	
GCF combination		1.0581		0.0560		0.0510		0.1041		0.0400	
Residual		0.3498		0.1178		0.1400		0.1437		0.0631	

*Bold p values indicate significant effects (p < 0.05).

Supplementary Table 5 Comparison of community-type specific responses to individual GCFs and GCF number. Results of linear (mixed effects) models testing the effects of the single GCF treatments (a) and GCF number (b) on aboveground biomass, species composition (PC1 and PC2 of PCA on species biomass proportions), diversity (Shannon index) and evenness of the transplanted-seedling and sown communities jointly. Community type (i.e. transplanted-seedling vs sown) was included as a fixed effect. Generally, we fitted linear mixed effects models but when inclusion of random effects did not improve the model, we used more parsimonious linear models. Significance of the fixed effects was assessed using log-likelihood ratios (χ^2 values) or F statistics, as indicated in the table.

		Total aboveground community biomass		Shannon index (species prop.)		Pielou evenness (species prop.)		Species composition (PC1)		Species composition (PC2)	
		(a) GCF-treatment model									
Fixed effects	df	F	p*	F	p	χ^2	p	χ^2	p	χ^2	p
GCF treatment	6	37.16	**< 0.001**	1.55	0.175	10.36	0.110	18.35	**0.005**	53.32	**< 0.001**
Community type	1	6.49	**0.013**	15.06	**< 0.001**	0.82	0.366	13.16	**< 0.001**	1.14	0.286
GCF:Community	6	1.1.56	0.171	0.77	0.595	3.72	0.715	7.44	0.281	30.95	**< 0.001**
Random effects		SD		SD		SD		SD		SD	
Plot		–		–		0.0135		0.0385		0.0571	
Residual		–		–		0.0856		0.1371		0.1379	
		(b) GCF-number model									
Fixed effects	df	χ^2	p	χ^2	p	χ^2	p	χ^2	p	χ^2	p
GCF number	1	3.81	*0.051*	28.72	**< 0.001**	35.82	**< 0.001**	0.098	0.792	1.54	0.214
Community type	1	29.23	**< 0.001**	53.04	**< 0.001**	0.29	0.593	17.49	**< 0.001**	0.18	0.674
GCF number:Community	1	3.10	*0.078*	5.90	**0.015**	3.27	*0.071*	4.21	**0.040**	3.69	*0.055*
Random effects		SD		SD		SD		SD		SD	
Plot		0.0523		0.0196		–		–		0.0175	
GCF combination		0.4944		0.1030		0.0241		0.0404		0.0737	
Residual		0.2679		0.2128		0.1156		0.1463		0.1336	

*Significant effects ($p < 0.05$) are shown in bold, marginally significant effects ($p < 0.1$) in italics.

Supplementary Table 6 Assessment of eutrophication contributions to GCF-number effects. Results of the linear mixed effects models used to disentangle the effects of eutrophication and GCF number, for all community variables that were affected by GCF number. To test whether GCF-number effects were driven by eutrophication effects, we added a binary eutrophication variable to include information on whether eutrophication was included in the respective GCF combination or not. GCF number, community type, eutrophication and the respective two-way interactions were included as fixed effects. Significance of the fixed effects was assessed using log-likelihood ratios (χ^2 values).

		Total aboveground community biomass		Shannon index (species prop.)		Pielou evenness (species prop.)		Species composition (PC2)	
Fixed effects	df	χ^2	p*	χ^2	p	χ^2	p	χ^2	p
GCF number	1	1.44	0.23	9.32	**0.002**	10.00	**0.002**	12.03	**< 0.001**
Community type	1	29.34	**< 0.001**	53.16	**< 0.001**	0.31	0.58	0.17	0.68
Eutrophication	1	49.79	**< 0.001**	0.51	0.48	4.58	**0.03**	0.04	0.85
GCF number:Community	1	3.39	*0.07*	3.01	*0.08*	0.33	0.56	0.54	0.46
Eutrophication:Community	1	0.41	0.52	0.43	0.51	3.55	*0.06*	3.23	*0.07*
GCF number:Eutrophication	1	0.004	0.95	0.05	0.83	0.11	0.74	4.36	**0.04**
Random effects		SD		SD		SD		SD	
Plot		0.0569		0.0127		0.0135		0.0217	
GCF combination		0.1499		0.1088		–		0.0553	
Residual		0.2679		0.2134		0.1141		0.1327	

*Significant effects ($p < 0.05$) are shown in bold, marginally significant effects ($p < 0.1$) in italics.

Supplementary Table 7 Single-GCF and GCF-number effects on the number of plants per species. Effects of single-GCF treatments and GCF number on the number of individuals per species present at harvest. Effects were assessed by fitting generalized linear mixed effects models. Significance of the fixed effects was assessed using log-likelihood ratios (χ^2 values).

(a) GCF-treatment model			
Fixed effects	df	χ^2	p*
GCF treatment	1	31.53	**< 0.001**
Community type	6	66.46	**< 0.001**
GCF:Community	1	12.89	**0.045**
Random effects		SD	
Species		0.9958	
**(b) GCF-number model**			
Fixed effects	df	χ^2	p
GCF number	1	12.99	**< 0.001**
Community type	1	594.09	**< 0.001**
GCF number:Community	1	0.13	0.719
Random effects		SD	
Pot		0.041	
GCF combination		0.322	
Species		0.925	

*Significant effects ($p < 0.05$) are shown in bold.

Supplementary Table 8 Single GCF treatments. Summary of the materials or equipment used to implement the single GCF treatments, and the intensity or concentration of each GCF. The table also lists the reference we used to choose the intensity or concentration of the GCF.

Treatment	Materials / Equipment	Intensity / Concentration	References
Warming	infrared-heating lamps	+1.99 (± 0.2) °C	IPCC [1] (RCP 4.5)
Eutrophication	Universol® blue (oxide) fertilizer	≙ 100 kg N ha^{-1}	FAOSTAT [2]
Soil salinization	NaCl	6 dS m^{-1}	Richards [3]
Light pollution	LED spotlights	24.5 lx	Bennie *et al.* [4]
Microplastic pollution	EPDM granules, 1 - 2.5 mm	1% (w/w)	Fuller and Gautam [5]
Fungicide accumulation	Landor® CT	1.5 µl pot^{-1}	Syngenta Agro GmbH, Landor® CT Produktinformationen

Supplementary Table 9 Study species. List of the nine study species, including the functional group, family and seed source.

Species	Family	Functional group	Seed source
Alopecurus pratensis L.	Poaceae	Perennial grass	Rieger-Hofmann GmbH
Lolium perenne L.	Poaceae	Perennial grass	Rieger-Hofmann GmbH
Poa pratensis L.	Poaceae	Perennial grass	Rieger-Hofmann GmbH
Sinapis arvensis L.	Brassicaceae	Annual forb	Rieger-Hofmann GmbH
Sonchus oleraceus L.	Asteraceae	Annual forb	Botanischer Garten Konstanz
Vicia sativa L.	Fabaceae	Annual forb	Revierberatung Wolmersdorf
Diplotaxis tenuifolia (L.) DC.	Brassicaceae	Perennial forb	Wild collected in Konstanz
Prunella vulgaris L.	Lamiaceae	Perennial forb	Rieger-Hofmann GmbH
Vicia cracca L.	Fabaceae	Perennial forb	Rieger-Hofmann GmbH

Supplementary Table 10 Germination information for the transplanted-seedling community.

Species	Sowing date	Germination Conditions
Poa pratensis	17/06/2020	Greenhouse; 16:8 hrs, day:night; ventilation windows opened at 21°C during day, and 18°C during night
Prunella vulgaris		
Alopecurus pratensis	22/06/2020	
Diplotaxis tenuifolia		
Lolium perenne		
Sonchus oleraceus[*]	22/06/2020; 29/06/2020	
Sinapis arvensis[†‡]	25/06/2020	Growth chamber; 9:15 hrs, 21°C:16°C, day:night; transferred to greenhouse on 02/07/20
Vicia cracca[†‡]		
Vicia sativa[†]		

[*]*Sonchus oleraceus* was sown twice to ensure a sufficient number of seedlings, since too few seeds germinated the first time.

[†]These species require short day conditions for germination.

[‡]There were not enough seedlings of *Vicia cracca* and *Sinapis arvensis* when the first transplanting was done and both species were re-sown on 6 July 2020.

Supplementary Table 11 GCF combinations used for the different GCF-number levels.

	GCF number																			
GCF treatment	0	1						2						4						6
Fungicide		■								■		■		■		■		■	■	■
Light pollution			■					■			■			■	■			■	■	■
Microplastic				■					■			■			■	■	■		■	■
Eutrophication					■				■				■	■	■		■	■		■
Salinization						■				■	■				■	■	■	■		■
Warming							■	■					■	■		■	■		■	■
Replicates†	6	6	6	6	6	6	6	6	6	6	6	6	6	6	6	6	6	6	6	6

†The transplanted-seedling and sown communities each had six replicates for each GCF combination. Hence, there were for each community type six replicates for the lowest (0), and highest (6) GCF-number levels, and 36 (6 × 6) replicates for the intermediate GCF-number levels (1, 2 and 4).

Supplementary Table 12 Factor identity and interaction contributions to GCF-number effects on the number of plants per species. Comparison of the hierarchical diversity-interaction models (Fig. 4) based on log-likelihood-ratio tests (χ^2 statistics, as indicated in the table), to assess individual factor and factor-interaction contributions to GCF-number effects on the number of plants per species. The diversity-interaction models were only run for response variables for which there were significant effects of increasing numbers of GCF.

**Transplanted-seedling Community**					
		Number of individuals			
Model (reference-model number)	DF	AIC	logLik	χ^2	p*
1 Null	-	926.31	-458.15	-	-
2 GCF identity (1)	5	**923.87**	-451.94	12.43 (+)	**0.03**
3 Separate pairwise GCF interactions (4)	10	**912.33**	-435.17	33.42 (+)	**<0.001**
4 Average GCF interactions (2)	1	925.75	-451.88	0.12	0.73
5 Additive GCF-specific interaction contributions (3)	5	911.03	-439.51	8.69	0.12
**Sown Community**					
Model (reference-model number)	DF	AIC	logLik	χ^2	p
1 Null	-	2409.7	-1199.9	-	-
2 GCF identity (1)	5	**2408.2**	-1194.1	11.54 (+)	**0.042**
3 Separate pairwise GCF interactions (4)	10	**2402.7**	-1180.3	27.09 (+)	**0.003**
4 Average GCF interactions (2)	1	2409.8	-1193.9	0.42	0.52
5 Additive GCF-specific interaction contributions (3)	5	2399.2	-1183.6	6.49	0.26

*Significant effects ($p < 0.05$) are shown in bold.

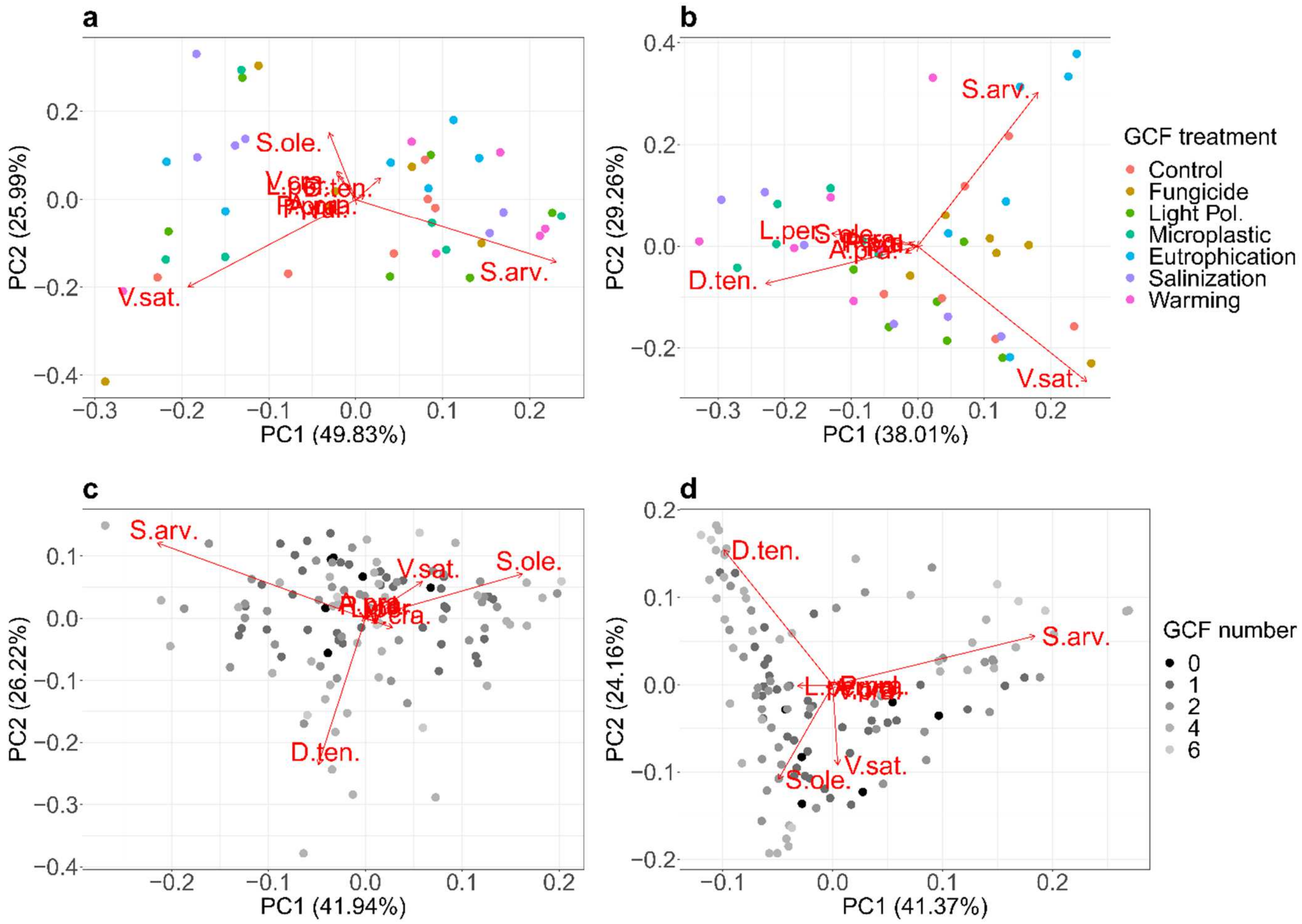

Supplementary Fig. 1 Single-GCF and GCF-number effects on species composition. Principal component analysis for transplanted-seedling (left panels) and sown (right panels) communities for GCF-number (c & d) and single GCF (a & b) effects based on species biomass proportions. Arrows indicate the different species. Differently colored points indicate the individual GCF factors or the GCF number. The species, abbreviated with the first letter of the genus and the first three letters of the species epithet were *Alopecurus pratensis, Diplotaxis tenuifolia, Lolium perenne, Poa pratensis, Prunella vulgaris, Sinapis arvensis, Sonchus oleraceus, Vicia cracca* and *Vicia sativa*.

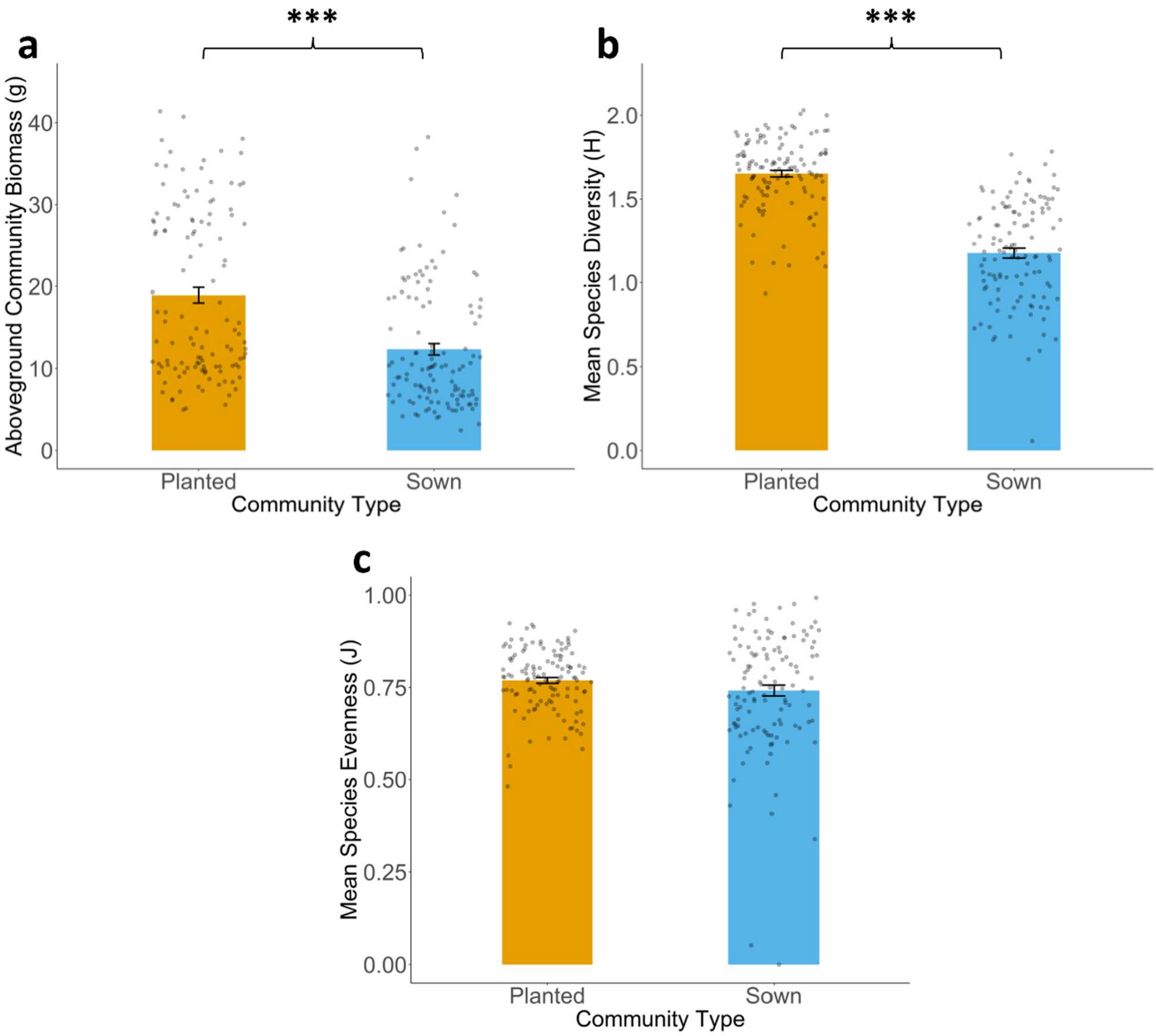

Supplementary Fig. 2 General comparison of transplanted-seedling and sown communities. Differences between both community types in terms of productivity (a; $p < 0.001$), species diversity (b; $p = 3.27e-13$), and species evenness (c; $p = 0.59$). The productivity, species diversity, and species evenness of the sown community were lower compared to the transplanted-seedling community (-34.7%, -28.8%, and -3.6%, respectively). Asterisks indicate significant differences between both community types ($p < 0.001$; Table S5). Error bars indicate standard errors. Differences between the community types were assessed with log-likelihood ratios (χ^2 values) or F statistics.

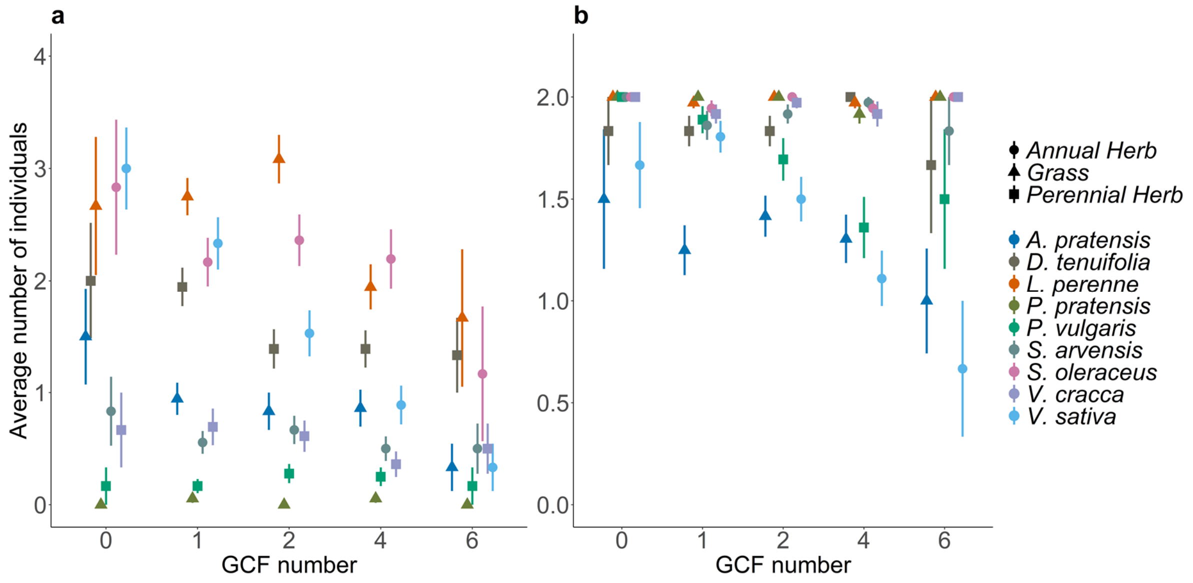

Supplementary Fig. 3 GCF-number effects on the number of plants per species in both community types. Average number of individuals of each species in the sown (a) and transplanted-seedling communities (b) at harvest under increasing numbers of co-acting factors. Colored symbols (circles, squares, etc.) represent the mean number of plants of each species in one mesocosm of one community type ($N = 6$ for GCF-number levels 0 and 6, respectively, $N = 36$ for GCF-number levels 1, 2, 4, respectively). Error bars represent standard errors. The species were *Alopecurus pratensis, Diplotaxis tenuifolia, Lolium perenne, Poa pratensis, Prunella vulgaris, Sinapis arvensis, Sonchus oleraceus, Vicia cracca* and *Vicia sativa*. Note the different y-axis scales.

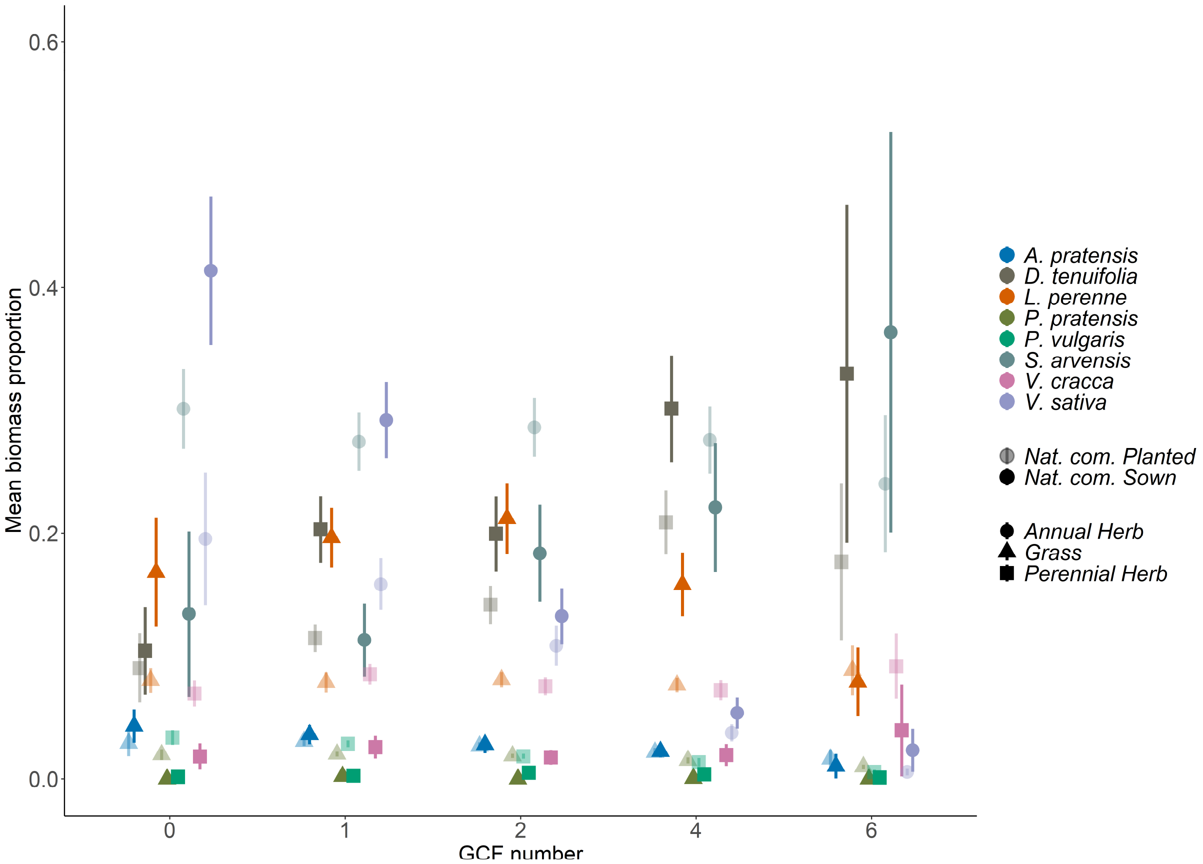

Supplementary Fig. 4 GCF-number effects on mean species biomass proportions. Biomass proportions of species in the transplanted-seedling (light colors) and sown (dark colors) communities in dependence of the number of simultaneously acting GCFs. Symbols represent mean values; symbol shapes indicate the functional group to which the species belongs and error bars represent standard errors. For each community type, $N = 6$ for GCF-number levels 0 and 6, respectively, $N = 36$ for GCF-number levels 1, 2, 4, respectively.

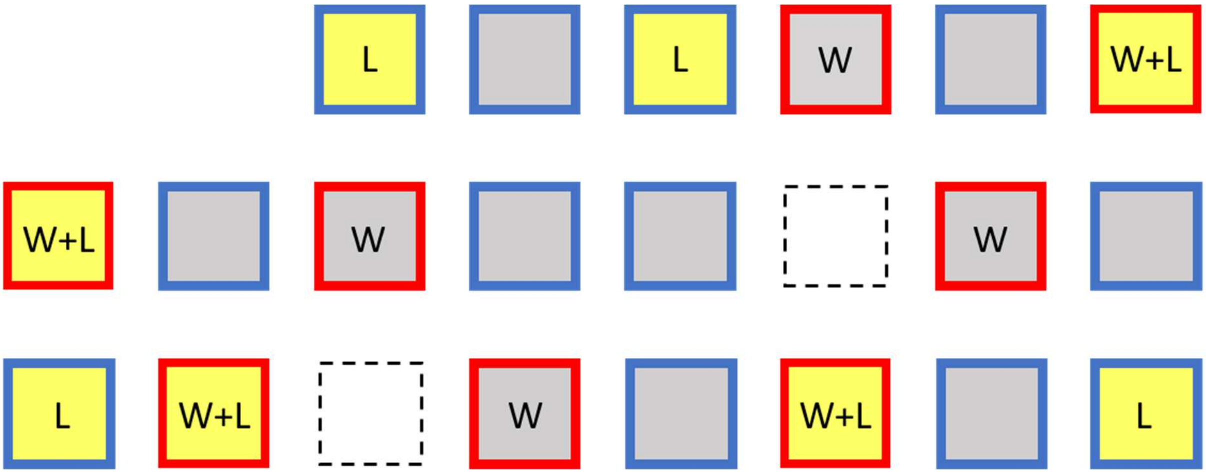

Supplementary Fig. 5 Schematic representation of the experimental area and the arrangement of the climate warming and light-pollution treatments. Each square indicates one 2 m × 2 m plot. Letters within boxes and colors denote the treatments of the respective plot. Yellow fillings indicate plots with light pollution (L), and grey fillings indicate plots without light pollution. Red borders indicate plots with warming (W), and blue borders indicate plots without warming. All plots, irrespective of the actual treatment or treatment-combination, were equipped with the same metal frames – either with or without infrared lamps – and non-functional dummy LED lamps or functional LED lamps, to ensure equal basic conditions (e.g., shading) for all plots. Empty boxes with dashed borders represent plots which were not used in the experiment.

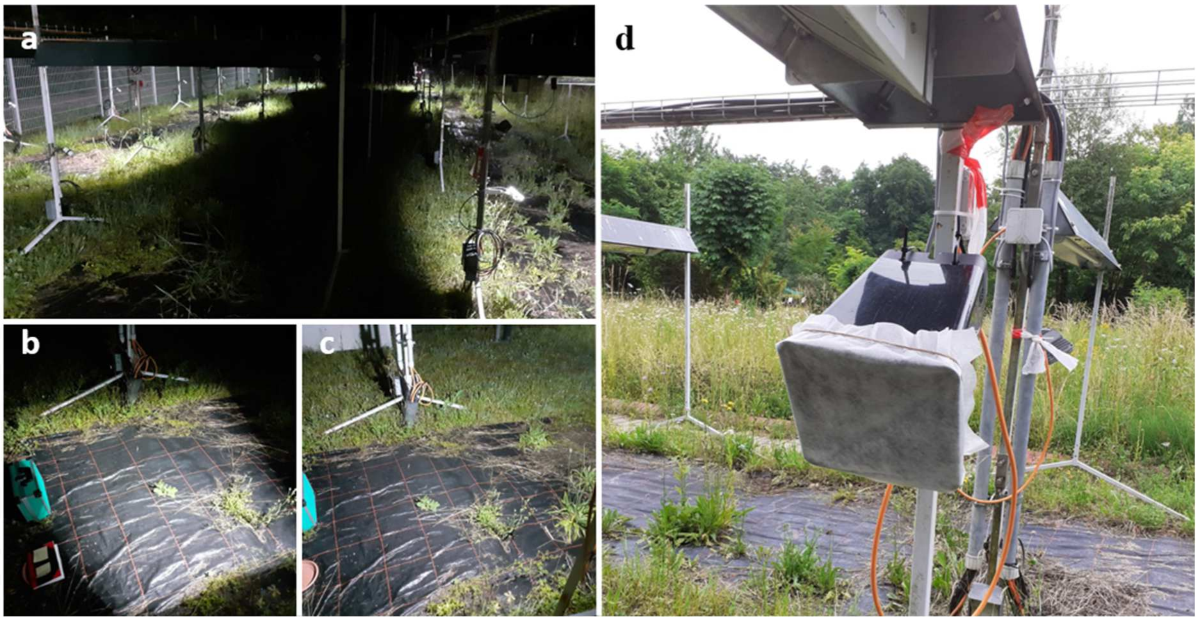

Supplementary Fig. 6 Photos illustrating the effectiveness of the light-pollution treatment. (a) To prevent illumination of plots without light-pollution treatment, the light-pollution plots were assigned to the outer plots (see Fig. S5), and spotlights were pointing away from the inner plots. (b, c) The effectiveness of using lampshades (black pot in d): (b) illuminated area of an LED lamp with lampshade, where illumination is restricted to the area of one plot; (c) illuminated area if we would have used LED lamps without lampshades. (d) LED lamp equipped with a lampshade (i.e. the black square pot) used to restrict the illuminated area, and which was covered with white tissue to reduce the light intensity from approx. 100 lx to a realistic average light-pollution level of approx. 24.5 lx at plant level.

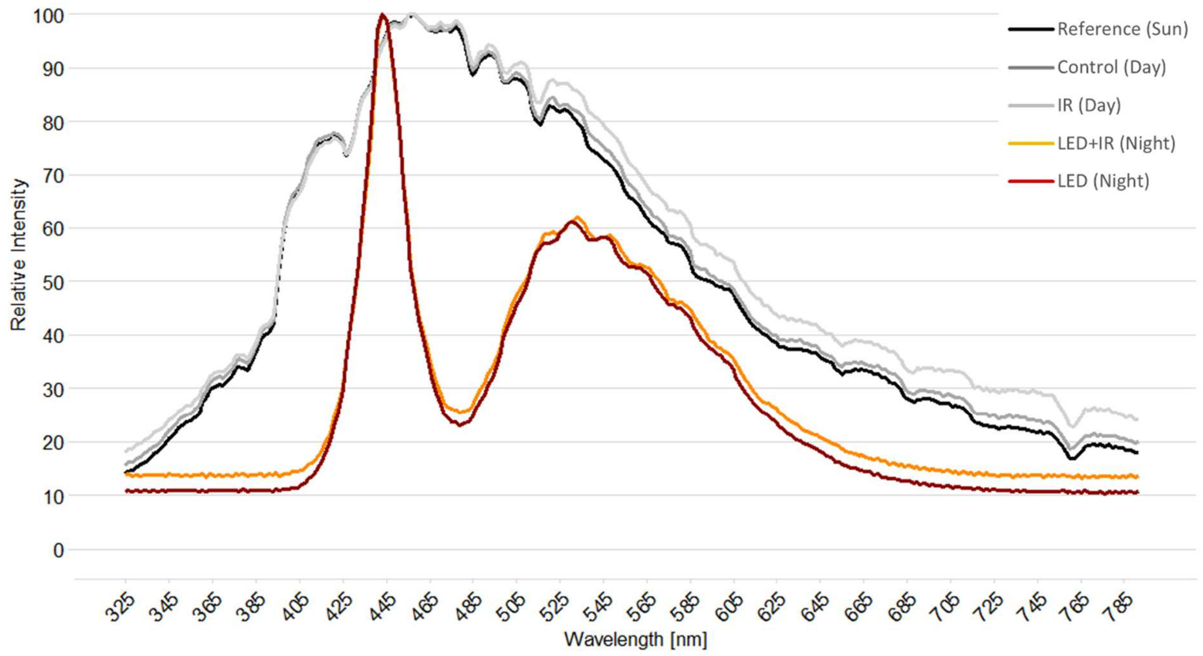

Supplementary Fig. 7 Comparison of natural and artificial light. Comparison of spectral composition of light available to plants during the day (black, light gray, dark gray), and night (red, orange). The spectral composition during the day is hardly altered by infrared (IR) lamps. Light emitted by LEDs shows two strong peaks, one in the blue range of the spectrum (maximum around 440 nm), and another one in the green range (around 530 nm). In addition, the light emitted by LEDs shows a marked gap in the transition area from the blue to the green part of the spectrum, constituting a major difference to sunlight.

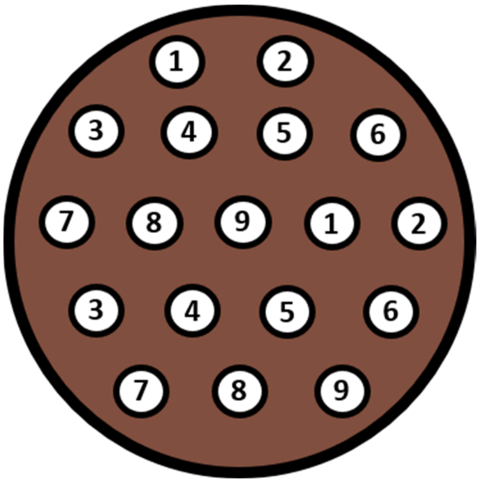

Supplementary Fig. 8 Schematic illustration of the positions of seedlings in the pots of the transplanted-seedling community. Each number indicates the position and identity of one of the nine species used. All 120 pots of the transplanted-seedling community had the species arranged in this way. Numbers refer to: 1 = *Poa pratensis*, 2 = *Sonchus oleraceus*, 3 = *Vicia cracca*, 4 = *Lolium perenne*, 5 = *Vicia sativa*, 6 = *Prunella vulgaris*, 7 = *Alopecurus pratensis*, 8 = *Sinapis arvensis*, 9 = *Diplotaxis tenuifolia*.

References

1. IPCC, *Climate change 2014: Synthesis report. Contribution of working groups I, II and III to the fifth assessment report of the intergovernmental panel on climate change*, ed. Core Writing Team, et al. 2014, Geneva, Switzerland.
2. FAOSTAT, *Fertilizer indicators*. 2020.
3. Richards, L.A., *Diagnosis and improvement of saline and alkali soils, No. 60*. 1954, Washington D.C.: U.S. Government Printing Office.
4. Bennie, J., et al., Ecological effects of artificial light at night on wild plants. *Journal of Ecology*, **104**(3), 611-620 (2016).
5. Fuller, S. and A. Gautam, A procedure for measuring microplastics using pressurized fluid extraction. *Environmental Science & Technology*, **50**(11), 5774-5780 (2016).
